# A Case of Recurrent Epiploic Appendagitis Treated With Conservative Management

**DOI:** 10.7759/cureus.27686

**Published:** 2022-08-04

**Authors:** Rachel V Christenson, Phuoc D Nguyen, Lincoln R Wallace

**Affiliations:** 1 Medical School, Des Moines University College of Medicine, Des Moines, USA; 2 Family Medicine, UnityPoint Health - Trinity Regional Medical Center, Fort Dodge, USA

**Keywords:** conservative vs surgical management, treatment of appendagitis, recurrent epiploic appendagitis, epiploic appendagitis, primary epiploic appendagitis (pea)

## Abstract

Epiploic appendagitis is a rare and commonly misdiagnosed cause of acute abdominal pain. Treatment for the initial presentation of epiploic appendagitis is conservative management with anti-inflammatory medications. There is no consensual treatment algorithm for recurrent appendagitis as some studies recommend conservative management and others recommend surgical excision. This case report will cover a patient who presented with recurrent epiploic appendagitis and was treated with conservative management.

## Introduction

Epiploic appendages are benign outpouches of peritoneal structures on the outer surface of the bowel wall. They are composed of adipose tissue and a vascular stalk [1]. When an epiploic appendage undergoes ischemic infarction by torsion or thrombosis of the appendage central vein it results in epiploic appendagitis. Epiploic appendagitis is up to four times higher in men and commonly occurs between the second and fifth decades of life [2]. They can occur in any portion of the colon but most commonly arise in the rectosigmoid colon. Possible risk factors for developing epiploic appendagitis include obesity and strenuous exercise [3,4]. 

Often, the first symptom of epiploic appendagitis is acute or subacute onset of lower abdominal pain. Clinical presentation includes constant, localized, non-migrating pain without fever, nausea, diarrhea, vomiting or rebound tenderness. In some patients, a mass can be palpated, and laboratory workup is usually within normal limits [2]. Due to the nonspecific symptoms, epiploic appendagitis can commonly be misdiagnosed as acute diverticulitis or acute appendicitis, leading to unnecessary surgeries, hospitalization, and antibiotic usage [1]. Abdominal computed tomography (CT) is diagnostic for epiploic appendagitis and ultrasound may be used if CT findings are unreliable [5]. Treatment for epiploic appendagitis starts with conservative management with anti-inflammatory medications (non-steroidal anti-inflammatory drugs [NSAIDs]) and a short course of opioids if needed for pain control [2]. Surgery (laparoscopic appendage excision) is indicated if symptoms do not improve with conservative management, if patients present with worsening symptoms, or if complications (eg, abscess, obstruction, intussusception) arise [2].

## Case presentation

A 22-year-old male with a BMI of 31.2 presented to the Emergency Department with abdominal pain that began one day before. The lower left quadrant (LLQ) pain had been constant since onset with exacerbation of pain reported with palpation. He rated his current pain as 7/10 in severity. During the visit the patient also reported a bloated sensation as well as a decreased appetite. He specified that he had eaten that day without an increase in pain, though he consumed a lesser amount. He had no other symptoms including nausea, vomiting, fever, nor any changes in bowel movements or urination. The patient had not noticed an abdominal or inguinal bulging/mass and had no recent injury or strenuous lifting. He had no evidence of ingestion of contaminated foods or water. He denied recent travel, use of antibiotics and had no abdominal surgical history. He treated the pain using Tums (calcium carbonate, antacid) and Gas-X (simethicone) prior to ED without significant changes.

**Figure 1:**
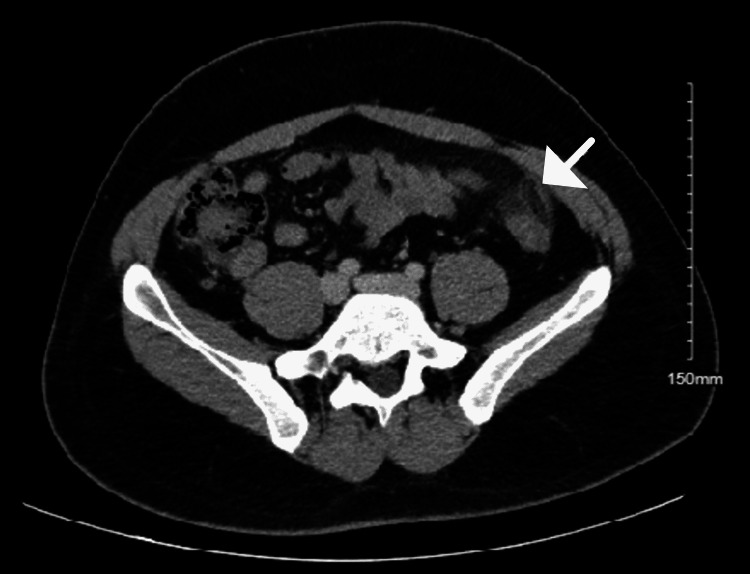
Contrast-enhanced axial section Abdomen and Pelvis CT scan noting epiploic appendagitis at the distal descending colon.

Laboratory workup was within normal limits with a mildly elevated white blood cell count and increased absolute neutrophils (Table 1). CT of the abdomen and pelvis with contrast reading (Figure 1) revealed an ovoid fat attenuation structure with an enhancing rim along the anterior margin of the distal descending colon. There was infiltration of the surrounding fat and a tiny amount of fluid in the left paracolic gutter. A small amount of free fluid in the dependent portion of the lower pelvis was noted. There were no colonic diverticulae. The appendix appeared normal. The final impression by the radiologist was an epiploic appendagitis at the distal descending colon. 

**Table 1:** The patient’s laboratory values /mL: per microliter mg/dL: milligrams per decilitre

	Patient's values	Reference Range
White blood cell	11,560/mL	4,500 - 11,000/mL
Absolute neutrophils	8,400 /mL	2,500 - 7,000/mL
Glucose	118 mg/dL	70 - 99 mg/dL

The patient was treated in the Emergency Department with 0.9% NaCl infusion, 30mg intravenous injection of ketorolac and 4mg intravenous injection of ondansetron HCl. A surgical consult was called and conservative management without surgery was suggested by the on-call surgeon. The patient improved with treatment and was released home with hydrocodone-acetaminophen, ibuprofen, ondansetron, and senna-docusate. 

Sixteen months after initial presentation, the patient returned to his primary care provider (PCP) for evaluation of recurrent pain in his left lower quadrant. The pain began earlier in the week and was fairly consistent, painful, and localized. Location was similar or the same as the previous episode that was worked up in the ED and diagnosed as an epiploic appendagitis. The patient stated that the first episode of epiploic appendagitis resolved and he has not had any issues prior to this episode. He denied fevers, chills, nausea, vomiting, stool changes or urinary symptoms. Physical exam revealed an abdomen that was flat, soft, nondistended, with positive bowel sounds in all four quadrants, with no masses or organomegaly. Pain was reproducible to deep palpation in the left lower quadrant, with no guarding or rebound tenderness.

**Figure 2:**
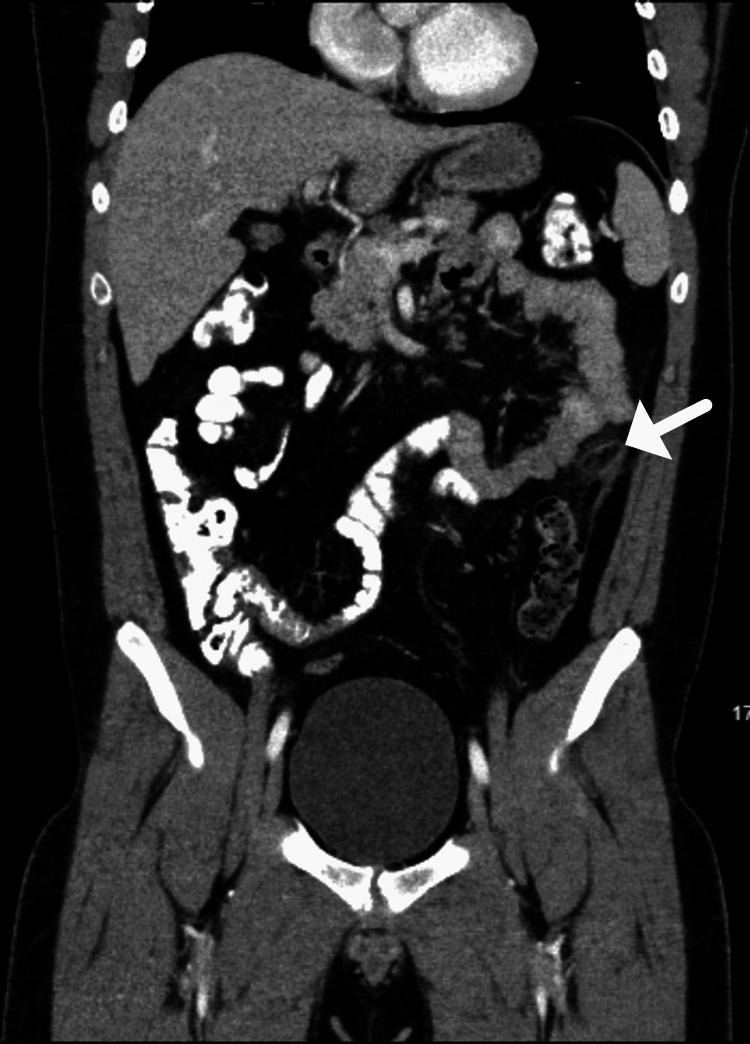
Contrast-enhanced coronal section Abdomen and Pelvis CT scan noting epiploic appendagitis at the mid descending colon.

A repeat CT of the abdomen and pelvis with contrast (Figure 2) was reported as follows: an ovoid fat attenuation structure along the anterolateral margin of the descending colon demonstrating mild surrounding mesenteric inflammation and adjacent colonic wall thickening. This appears similar to previous CT and is most suggestive of epiploic appendagitis. The appendix is normal. No pneumatosis, free fluid or free air. Final impression by the radiologist was epiploic appendagitis at the mid descending colon.

Due to the rarity of a recurrent epiploic appendagitis, the PCP consulted general surgery. The on-call surgeon suggested that even with the recurrence it would remain a self-limiting process and as such should be treated with anti-inflammatories and analgesics as needed. If it did not resolve on its own the patient should return to the hospital clinic or call for guidance. The patient was prescribed hydrocodone-acetaminophen and told to report back if the pain did not subside. The patient did not follow up. 

## Discussion

Epiploic appendagitis is a relatively uncommon inflammatory condition of fat-filled appendages of the colon. It occurs at a frequency of 1.3% and has an incidence of 8.8 per million people every year [6] although this number may be increasing due to better diagnostic imaging with contrast enhanced CT. The inflamed outpouching ranges from 0.5 to 5 cm in the largest dimension and are located along the serosal surface of the colon. They are commonly found in the sigmoid and transverse colon but may occur anywhere throughout the colon [1]. Each appendage is supplied by arteries that branch from the vasa recta longa and one central draining vein. It is hypothesized that the epiploic appendages serve as a blood reservoir, assist in immunity and colonic absorption, and have a protective role for intestinal vessels during colon distension or collapse [1,7]. The incidence is more prevalent in men than women. Epiploic appendagitis has an association with obesity, hernias, and strenuous exercise [1]. In a study of 208 cases of epiploic appendagitis, 73% of cases were caused by torsion of the appendage leading to obstruction of vascular flow and aseptic necrosis. Eighteen percent of cases were due to hernia incarceration, 8% due to intestinal obstruction, and less than 1% were due to an intraperitoneal loose body [8]. 

Patients with epiploic appendagitis often present with a constant, dull and localized, non-migrating pain in the lower abdomen. Most patients report pain in the left abdomen but there are also reports of right abdominal pain. Patients typically present afebrile and deny nausea, vomiting, diarrhea, or rebound tenderness. Laboratory workup is generally within normal limits. Similarly, our patient presented with lower left quadrant pain and minimal other symptoms for the first and second episode of epiploic appendagitis [7]. 

The diagnosis of epiploic appendagitis is challenging due to the nonspecific nature of the disease. It can often be misdiagnosed as acute diverticulitis or acute appendicitis because of overlapping of certain symptomology. These misdiagnoses lead to unnecessary antibiotic use, hospitalizations and surgeries [1]. The use of abdominal computed tomography is the diagnostic choice, but can be supplemented with ultrasound and doppler studies if the computed tomography is non-diagnostic [5].

**Figure 3:**
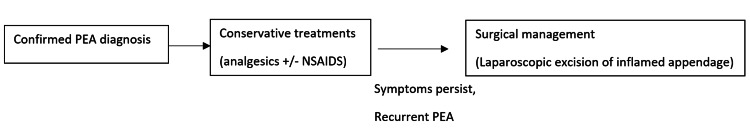
Epiploic appendagitis proposed treatment algorithm PEA: Primary epiploic appendagitis, NSAIDs: Non-steroidal anti-inflammatory drugs

**Figure 4:**
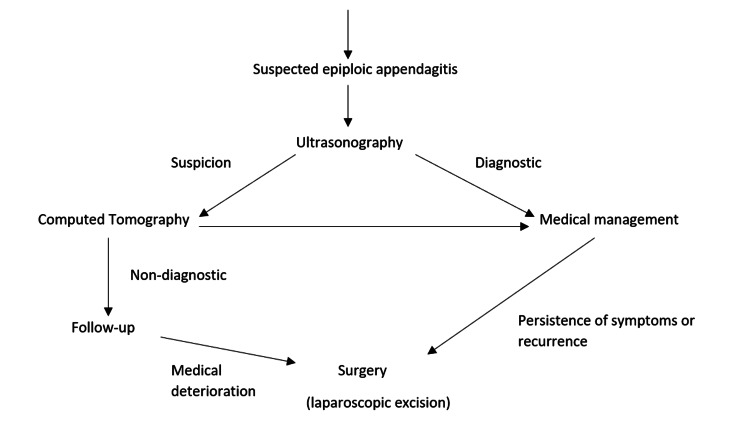
Proposed algorithm for patients with suspected epiploic appendagitis

Current articles recommend conservative management with or without NSAIDs as the first choice of treatment for primary epiploic appendagitis. Symptoms typically resolve within several days to weeks of conservative management but there is a high rate of recurrence [2]. In the case of recurrent epiploic appendagitis, Giannis et al. recommend surgical management (laparoscopic appendage excision) (Figure 3). It should be noted that CT findings of epiploic appendagitis may persist for several months and providers should be educated on the residual imaging findings to avoid misdiagnosis [2]. A case series and literature review by Ozdemir et al. suggests a different approach to treatment of recurrent epiploic appendagitis, that surgical management should be recommended only in cases of clinical and laboratory deterioration (Figure 4) versus UpToDate's current guidelines of surgical management for recurrent appendagitis [9,10]. This case follows Ozdemir's suggestion and the patient was treated conservatively for both primary and recurrent epiploic appendagitis [9].

In our case, surgery was consulted for the primary and recurrent epiploic appendagitis. In both instances, surgery was not recommended, and the patient was treated with conservative management. The surgeon did not recommend laparoscopic excision of the recurrent epiploic appendagitis because even with recurrence it should remain a self-limiting disease. 

While there is overall agreement that if CT is done properly for primary epiploic appendagitis and the patient is stable, then the patient can be treated with conservative management and surgery consultation may not be needed. While ultrasound is a viable alternative if ionizing radiation is contraindicated, CT is a superior diagnostic tool. This allows for a PCP or ER physician to manage appendagitis without the need for surgical consult. There is no evidence-based standardized care plan from randomized control trials or meta-analysis for patients with recurrent appendagitis and current management is up to the surgeon. A standardized care plan would reduce the number of unnecessary surgical consults, provide evidence-based treatment to improve patient outcomes, and promote consistency of care.

Antibiotics are not recommended for treatment of epiploic appendagitis. In a study by Choi et al., 30 out of 31 patients with epiploic appendagitis were treated with antibiotics [11]. Inappropriate and excess administration of antibiotics may be the result of clinicians not being familiar with epiploic appendagitis and the lack of a standardized treatment algorithm.

Epiploic appendagitis treated conservatively can have complications including abscess development, gastrointestinal obstruction, and intussusception. Patients should be advised to seek medical care if experiencing worsening symptoms [2]. This may be avoided by a standardized treatment algorithm that includes a concrete workup and treatment of epiploic appendagitis complications. 

We suggest further studies to investigate the rate of epiploic appendagitis recurring in the same location. This may affect surgical management recommendations. There may also be a benefit in researching morbidity and mortality of epiploic appendagitis complications after conservative management. 

## Conclusions

Here we present a case of recurrent epiploic appendagitis managed by medical therapy. We conclude by pointing out the need for a consensual treatment algorithm for all physicians and surgeons to follow. There have been many proposed algorithms regarding treatment for epiploic appendagitis, but it is often up to the discretion of the surgical and medical management team. In this case, the plan deviated from the proposed protocols by the suggested withholding of definite surgery after the recurrent episode. 
